# 11-GHz waveguide Nd:YAG laser CW mode-locked with single-layer graphene

**DOI:** 10.1038/srep11172

**Published:** 2015-06-08

**Authors:** Andrey G. Okhrimchuk, Petr A. Obraztsov

**Affiliations:** 1 International Center of Laser Technologies, D. Mendeleyev University of Chemical Technology of Russia, 9 Miusskaya Square, Moscow 125047, Russia; 2 Fiber Optics Research Center of RAS, 38 Vavilova Str., Moscow 119333, Russia; 3 Prokhorov General Physics Institute of RAS, 38 Vavilova Str., Moscow 119333, Russia

## Abstract

We report stable, passive, continuous-wave (CW) mode-locking of a compact diode-pumped waveguide Nd:YAG laser with a single-layer graphene saturable absorber. The depressed cladding waveguide in the Nd:YAG crystal is fabricated with an ultrafast laser inscription method. The saturable absorber is formed by direct deposition of CVD single-layer graphene on the output coupler. The few millimeter-long cavity provides generation of 16-ps pulses with repetition rates in the GHz range (up to 11.3 GHz) and 12 mW average power. Stable CW mode-locking operation is achieved by controlling the group delay dispersion in the laser cavity with a Gires–Tournois interferometer.

Recently the development of ultrafast mode-locked (ML) lasers with high repetition rates have attracted great attention because of a growing number of applications. In particular, laser sources delivering stable trains of pico- or sub-picosecond pulses with repetition frequencies greater than 1 GHz are of special interest. This is due to the possibility for use of these types of lasers as generators of stable frequency combs, which are invaluable tools in such applications as optical clocks and frequency metrology^1^. Apart from frequency metrology, these lasers are prospective for applications in high-precision spectroscopy^2^, biophotonics^3^, ultrafast optical sampling^4^, non-linear microscopy^5^, telecommunications^6^, and in fundamental physics. To date, there are several approaches to create GHz-repetition rate ultrafast laser sources including mode-locked solid-state lasers, fiber lasers, operation with different kinds of saturable absorbers, semiconductor ridge-waveguide lasers^7^, and VECSELs^8^. However, one of the simplest and most reliable designs of GHz mode-locked lasers is based on the use of a short two-mirror cavity completely or almost completely filled with the dielectric gain medium (i.e. laser crystal or rare-earth doped glass)^9^. An important condition for achievement of a stable mode-locking regime is operation in a fundamental transversal mode with a stable transverse profile and without addition of any higher-order modes. Because of the thermal lens effect and the fact that the single-transversal mode generation regime is detrimental to the maximum output power and the generation of a pure fundamental mode with a stable size, it is hard to maintain in practice. In particular, the problem of single transverse mode generation is especially difficult to solve in lasers with short cavities, and consequently for ultra-high repetition rate lasers.

In waveguide solid-state lasers, as well as in fiber lasers, the thermal lens effect is suppressed by external boundary conditions on the transverse dimension of the field profile through the transverse refractive index profile. Higher-order modes are usually supressed by a proper design of the refractive index profile. For example, femtosecond pulses with pulse repetition rates of several GHz and average output power up to 27 mW were recently obtained in Er^3+^fiber lasers mode-locked with SESAM and carbon-nanotubes operating at 1.5 μm^10^,^11^,^12^. More recently, 3 GHz mode-locking of a Yb^3+^fiber laser was reported^13^. The short-cavity waveguide lasers mode-locked with SESAMs producing pulses at repetition frequencies in a range from 400 MHz to 15 GHz were reported^14^,^15^,^16^. Recently 1.5-GHz and 7.8-GHz Q-switch mode-locking was achieved in ytterbium-doped and thulium-doped quasi-monolithic waveguide lasers with graphene-based saturable absorbers^17^,^18^. However, because of high waveguide propagation losses and the large modulation depth of the saturable absorber, the authors were not able to achieve CW mode-locking.

Because of the short resonator length of the mode-locked lasers operating at GHz pulse repetition frequencies, the output pulse energy is rather limited. In passively mode-locked lasers operating without elements controlling the cavity dispersion, the low pulse energy leads to Q-switch instabilities disturbing the phase-lock between modes and therefore limiting the opportunities to achieve CW ML regime^19^. Recently, the way to avoid such instabilities through generation of solitons under an additional condition when the gain width spectrum is compatible with the pulse spectral width^16^,^19^,^20^ has been shown. In such circumstances, a negative feedback arises between the soliton energy and the overall pulse gain that in turn leads to a highly stable mode-locking regime for soliton formation in a two-mirror resonator with a length of a few mm, the latter supplemented with a Gires–Tournois interferometer, which provides the appropriate negative group delay dispersion (GDD) for round trip in the cavity^16^,^20^,^21^.

Recently continuous wave mode-locking with SESAM saturable absorber has been demonstrated in a short-cavity waveguide laser based on a phosphate glass IOG-1 doped with Y_2_O_3_ (12 wt.%)^15^. The waveguide was formed by ion exchange method, while GDD was controlled with a Gires–Tournois interferometer. However implementation of the SESAM, that is a HR mirror, necessitates the combination of pumping and output beams on another mirror, which complicates an optical scheme. In this letter, we employed a saturable absorber based on single-layer graphene deposited directly on the output coupler mirror, making the optical scheme more compact. The waveguide was inscribed in the crystal with a femtosecond laser beam. This method has advantages over other methods of forming the waveguides in providing higher flexibility to produce waveguides with different architectures of refractive index profile, relative simplicity, and less time-consuming process of the waveguide formation^22^,^23^,^24^,^25^,^26^.

A Q-switch mode-locking operation with GHz pulse repetition rates has been recently demonstrated in ultrafast laser-inscribed waveguide lasers^17^,^18^. In this letter, we report the first demonstration of stable multi-GHz repetition rate CW mode-locking of a direct laser-written waveguide laser operating with single-layer graphene saturable absorber.

## Methods: Waveguide and saturable absorber fabrication

**Figure 1:**
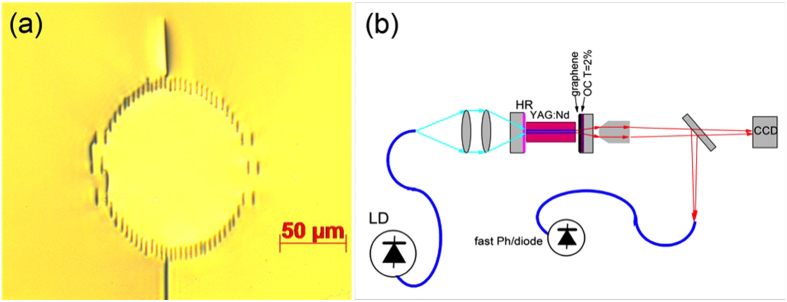
(**a**) Microscopic end view of the tabular cladding waveguide in the YAG:Nd crystal; (**b**) Laser setup. LD is a fiber coupled laser diode operating at 809 nm, fast Ph/diode is a photodiode with 12 GHz bandwidth, CCD is the beam profiler.

The tubular cladding waveguide in YAG:Nd crystal was fabricated with an ultrafast laser-inscription method using an amplified Ti:Sa laser producing 110-fs pulses with 1-kHz repetition rate at 800 nm central wavelength. The cladding of waveguide was formed by 66 tracks with depressed refractive index. The estimated laser-induced refractive index change in the track was as high as 2.5 × 10^−3^ 23. The microscopic bright field image of the fabricated waveguide is presented in Fig. 1(a). The waveguide core was slightly elliptical with vertical and horizontal axes lengths of 120 μm and 107 μm, respectively. The inscribed waveguide provided propagation of several transversal modes at 1.06 μm.

The saturable absorber was formed by direct deposition of a single-atom thick graphene layer on the output coupler mirror. Single-layer graphene was prepared using the chemical vapor deposition (CVD) method. The single-layer graphene film was grown on the 10 × 50 mm^2^, 50 μm-thick copper (Cu) foil using a cold-wall chemical vapor deposition (CVD) setup^27^. The foil was first heated by electrical current up to 850^o^–1050^°^ for 2 minutes in CH_4_/H_2_ atmosphere and then instantaneously cooled down to the room temperature by switching off the heating current applied to the foil. To remove the grown film from the Cu foil and to transfer the graphene to the target laser mirror, a chemical etching in 0.1 g/mL FeCl_3_ for 24 hours was performed. Before placing the sample in the etchant, one side of the foil with synthesized graphene was covered with polymer. After the Cu foil had been completely etched, the free-standing graphene film was washed in distilled water to remove traces of the FeCl_3_ etchant and then transferred onto the mirror. The good crystalline quality and one-atom thickness of the prepared graphene film were confirmed by Raman and optical transmission spectroscopy measurements^28^.

## Lasing experiments and results

The experimental setup is shown in Fig. 1(b). Two flat mirrors produce the feedback in the waveguide laser cavity. The dichroic back mirror (HR at 1064 nm and T = 99% at 809 nm) and the output coupler (T = 2% at 1064 nm) were mounted on angle-tunable translation stages end to end with the YAG:Nd crystal. The graphene film was deposited directly onto the dielectric coating of the output coupler. A wedge-shaped substrate was used for the output coupler in order to avoid the etalon effect. The distances between the crystal end-facets and the cavity mirrors were adjustable in the range 50–1000 μm. In the experiments, the 6-W laser diode operating at 809 nm coupled with a multimode quartz fiber (NA = 0.15, core diameter 200 μm) was employed as a pump source. After passing through the optical fiber, the pump beam was focused into the waveguide through a double-convex lens condenser. The output end of the waveguide was imaged with a 50-mm focus lens onto the CCD camera of a Spiricon beam profiler or an input of SMF-28 single-mode optical fiber coupled either with an ultrafast 12-GHz NewFocus 1554-B photodiode connected to a 16-GHz Tektronix TDS-DPO-71604C wide-band oscilloscope or with a high-resolution spectrum analyzer (Ando AQ6317B).

**Figure 2:**
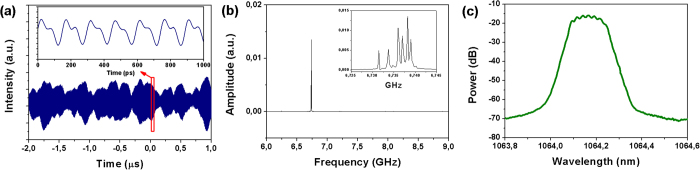
(**a**) Trains of output pulses obtained without GDD control. Two different timescales are shown: 3 μs and 1 ns (inset); (**b**) Radio-frequency spectrum; (**c**) Optical spectrum.

We performed two series of experiments with waveguides lengths of 11 mm and 6.7 mm and differing by of end-facet coatings. The first series of experiments was performed using an 11-mm long waveguide with antireflection-coated end facets. With the graphene saturable absorber (GSA) the laser was always operating in a mode-locking (ML) regime without tending to produce nanosecond envelopes of pulses related to Q-switching. However, as shown on Fig. 2 (a), on a microsecond time scale there were periodic amplitude oscillations resulting in significant timing jitter and appearance of satellite peaks in a frequency-domain spectrum (see Fig. 2 (b)). Most probably this behavior was related to the appearance of two or several pulses simultaneously circulating in the resonator.

In the second series of experiments we employed a nearly on-half shorter waveguide cavity with a length of 6.7 mm and control of the group delay dispersion (GDD). The pump end of the waveguide was covered with antireflection coating while the output end was uncoated. The uncoated waveguide end together with the output high-reflection mirror (***R***_***OC***_ = 98%) being the equivalent of an intracavity Gires–Tournois interferometer generates tunable GDD. The output mirror with graphene (GSA) was mounted onto the combined manual- and piezoelectric-driven micropositioning stage, allowing coarse and precise adjustment of the air gap size ***L***_***GTI***_ between the waveguide end and the output mirror covered with graphene (GSA) and therefore to control the group delay dispersion GDD in a wide range from −8 ps^2^ to 8 ps^2^.

**Figure 3:**
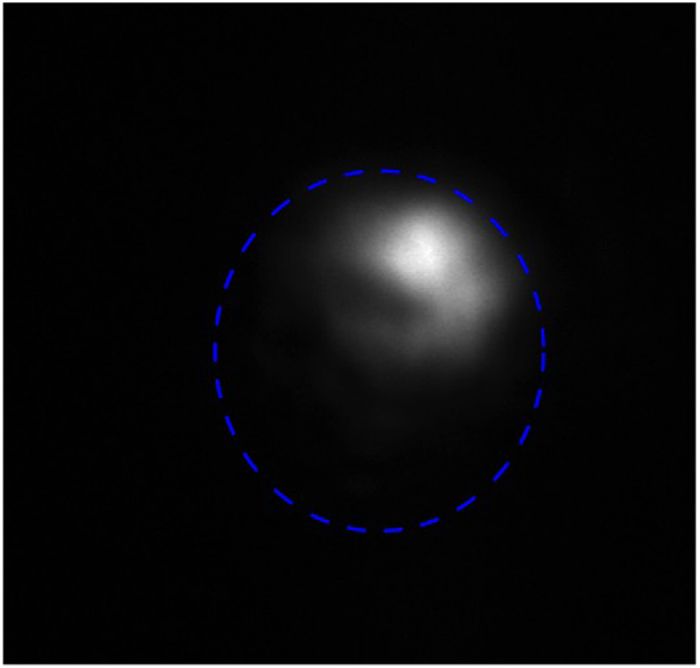
Waveguide laser mode profile taken at the output coupler for optimized ML. The blue dashed line denotes the waveguide core boundary, which is of 107 × 120 μm size.

**Figure 4:**
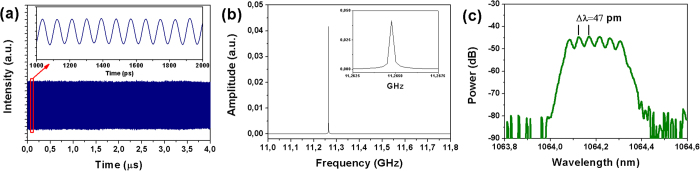
(**a**) Trains of CW mode-locked pulses in the cavity with GDD control on two different timescales: 4 μs and 1 ns (inset); (**b**) Radio-frequency spectrum; (**c**) Optical spectrum, resolution 0.01 nm.

We found that the intensity profile of the output laser spot crucially depends both on the mirror angle adjustments and on the distance between the mirrors and the waveguide. However nearly single mode CW lasing was achieved by angle tuning and slight adjustment of the positions of the cavity mirrors The intensity distibution of the output laser spot taken with a Spiricon laser beam profiler is shown in Fig. 3. The mode diameter at 1/e^2^ was found to be 54 μm. The threshold pump power was weakly dependent on the air gap size up to 2 mm and the lasing was always reached around pump power of 0.5 W while the maximum average output power ***P***_***out***_ of 12 mW at 1064 nm was achieved at a pump power of 3 W. Regardless of the air gap size and the pump power, the laser was always operating in the pulsed regime producing one or several overlapped trains of mode-locked pulses with typical periods of ~100 ps (repetition rate in range of 10–12 GHz). The stable CW mode-locked operation was achieved by adjustment of the air gap size ***L***_***GTI***_. It is important to note that we distinguished two types of adjustments leading to a stable CW ML regime, coarse and fine, on a few mm scale and hundreds of nm, respectively. The coarse tuning is related to the selection of transverse modes in the cavity while the fine tuning affects mostly the GDD. We found that the stability of CW ML strongly depends on both types of tuning and the best results were obtained when***L***_***GTI***_ was set to about 1 mm while the stability of ML demonstrates periodic dependence on the ***L***_***GTI***_ with period of around 260 nm. An analogous periodicity was observed in the optical spectrum (see Fig. 4(c)). As a result of the coarse and fine tunings, a stable train of ultrashort single pulses with 11.26 GHz pulse repetition frequency corresponding to the cavity round trip and minor (less than 5%) amplitude modulation was obtained. The obtained oscillogram and frequency domain spectrum are shown in Fig. 4 (a,b), respectively.

**Figure 5:**
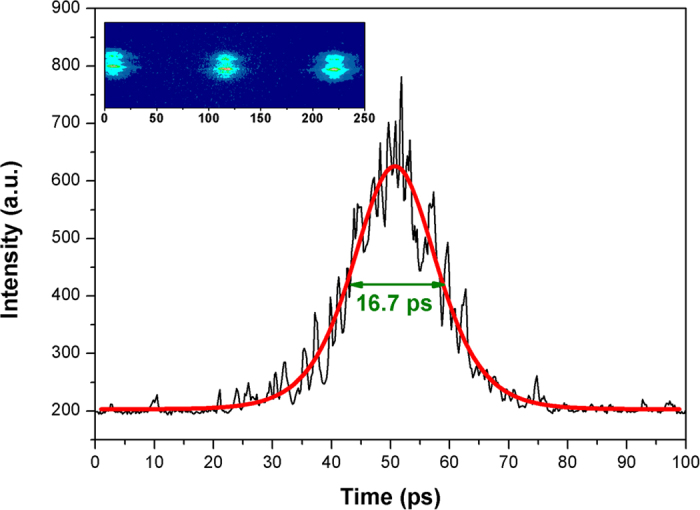
The measured streak-camera trace and sech^2^ fitting.

As one can see from the output spectrum profile (Fig. 4 (c)), six individual longitudinal modes were clearly resolved and were separated by a mode spacing of ~11 GHz. The laser output pulse energy (~1 pJ) was insufficient to permit measurement of the pulse duration by autocorrelation method so we employed a high-speed optoelectronic streak camera PS-1/S1^29^ with a 1-ps temporal resolution. The measured streak-camera trace and corresponding sech^2^(t) fit are shown in Fig. 5. The measured pulse duration was found to be ***τ***_***1/2***_ = 16.7 ps (FWHM).

## Discussion

The developed Nd:YAG waveguide laser produces stable combs of short pulses with period equal to the waveguide cavity round-trip time, and an optical spectrum containing clearly resolved equally spaced individual modes with the beat note corresponding to the cavity round trip time. Control of GDD in the cavity plays a crucial role to establish stable and self-starting operation in the mode-locking regime. Such behavior is typical for the CW ML regime. Possible mechanisms of longitudinal mode synchronization in the waveguide cavity are as follows: i) the formation of the classical Schrödinger soliton under conditions with net anomalous (negative) GDD for round trip in the cavity; ii) chirped solitary pulse (CSP) formation in the cavity with net normal (positive) GDD. To determine the particular physical mechanism of the obtained mode-locking regime, we performed numerical analysis of the parameters of our waveguide laser. We analyzed the stability of the CSP regime of our laser following the approach developed in Ref.^30^. According to definitions given in this paper, the dimensionless pulse energy 
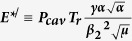
, circulating in the cavity, and parameter ***b*** **≡** ***αγ/β***_***2***_***ςμ*** (where: ***P***_***cav***_is the average intracavity power; ***P***_***cav***_*** = P***_***out***_***/(1-R***_***OC***_); ***T***_***r***_ is round-trip time of the cavity;***α*** is squared inverse transmission bandwidth; **γ** is the self-phase modulation (SPM) coefficient: for YAG:Nd, 1064 nm ***α*** = 10^−24^ s^2^, **γ** = 2*10^−6^ W^−1^; ***β***_***2***_ is the coefficient of GDD; ***ζ*** = ***1/I***_***s***_***S*** = 0.05 W^−1^ is inverse saturation power of GSA; ***I***_***s***_ = 0.87 MW is saturation intensity of graphene for the double-pass case^31^,^32^; ***S = ***2.3*10^−5^ cm^2^ is mode area; **μ** = 5.6*10^−2^ is the low signal loss coefficient of an ideal GSA for double pass) define the stability region for CSP in the E^*/^ - ***b*** master diagram. This region is defined by a condition that the gain does not exceed unsaturated total loss (the loss of GSA **μ** plus other non-saturable losses), and oscillations are only due to the dynamical drop of absorption in the GSA when a pulse with an appropriate energy encounters this element. A priori we do not know the GDD coefficient ***β***, so it is convenient to estimate a parameter 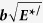 that is independent of ***β***, but which allows an understanding of whether the laser falls into the CSP stability region. Under our experimental conditions it can be calculated that 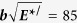, which is obviously inconsistent with the stability region for CSP because the calculated value of this product is very large^30^, Fig. 1. Therefore the mode-locking regime in the cavity with the positive (normal) dispersion cannot be realized under our experimental conditions.

Now we analyze the probability to establish mode-locking in the cavity with negative GDD due to formation of a Schrödinger soliton. Peak pulse power in the cavity can be estimated by the equation:


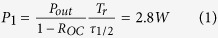


where ***τ***_***1/2***_ is FWHM pulse duration (measured to be 16.7 ps). Then estimating the net cavity GDD, which is necessary for the fundamental soliton propagation, by the equation^33^:


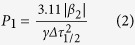


we arrive to the GDD value ***β***_***2***_ = –513 fs^2^.

**Figure 6:**
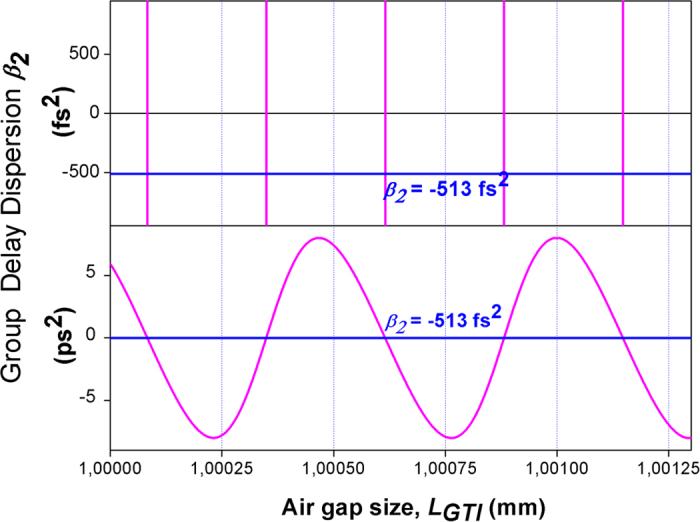
Dependence of total GDD in the laser cavity upon air gap size in GTI (magenta solid line), and level of GDD necessary for the fundamental Shrödinger solitons (***β***_2_ = −513 fs^2^, blue solid line).

The theoretical dependence of total GDD, consisting of contributions of GTI GDD and material GDD of YAG crystal, upon air gap size ***L***_***GTI***_ is shown in Fig. 6. One should note that the material dispersion in Nd:YAG crystal (850 fs^2^) is small in comparison with GDD which could be provided by the intracavity interferometer. The level of GDD necessary for formation of fundamental solitons is also shown in Fig. 6. Intersections of the dependencies, shown in Fig. 6, correspond to air gaps values optimal for stable mode-locking. In the experiment, because of technical reasons, we were unable to measure the air gap with sub-micrometer accuracy. However we observed pronounced periodic dependence of the ML stability on the air-gap size ***L***_***GTI***_, where the stable mode-locking regime reproduces itself with a period of approximately 260 nm, corresponding to a periodicity of coinciding of the total GDD with the value ***β***_***2***_ = –513 fs^2^ corresponding to solitons formation. Therefore the mechanism related to formation of a classical Schrödinger soliton during the cavity round trip is responsible for the obtained CW mode-locking.

## Conclusions

We reported a miniature diode-pumped ultrafast waveguide laser delivering 16 ps pulses at up to 11.5 GHz repetition rate and 12 mW average power at 1064 nm central wavelength. The laser is based on a depressed cladding waveguide fabricated in Nd:YAG by femtosecond direct laser writing. A single-layer graphene saturable absorber provides stable self-starting CW mode-locking. Tunable negative group velocity dispersion introduced by the intracavity Gires–Tournois interferometer provides stabilization of the mode-locking regime as well as precise adjustments of pulse repetition rate. The proposed approach of achieving stable CW mode-locking is based on the combined use of a waveguide architecture, a broadband graphene saturable absorber, and GDD control. The design is not limited to YAG:Nd as an active medium and can be applied in the development of compact waveguide lasers operating at GHz repetition rates based on other crystals for operation in a broad range of wavelengths.

## Additional Information

**How to cite this article**: Okhrimchuk, A. G. and Obraztsov, P. A. 11-GHz waveguide Nd:YAG laser CW mode-locked with single-layer graphene. *Sci. Rep.* **5**, 11172; doi: 10.1038/srep11172 (2015).
